# Fuzzy clustering of CPP family in plants with evolution and interaction analyses

**DOI:** 10.1186/1471-2105-14-S13-S10

**Published:** 2013-10-01

**Authors:** Tao Lu, Yongchao Dou, Chi Zhang

**Affiliations:** 1 School of Biological Sciences, Center for Plant Science and Innovation, University of Nebraska, Lincoln, Nebraska, USA

## Abstract

**Background:**

Transcription factors have been studied intensively because they play an important role in gene expression regulation. However, the transcription factors in the CPP family (cystein-rich polycomb-like protein), compared with other transcription factor families, have not received sufficient attention, despite their wide prevalence in a broad spectrum of species, from plants to animals. The total number of known CPP transcription factors in plants is 111 from 16 plants, but only 2 of them have been studied so far, namely TSO1 and CPP1 in *Arabidopsis thaliana *and soybean, respectively.

**Methods:**

In this work, to study their functions, we applied the fuzzy clustering method to all plant CPP transcription factors. The feature vector of each protein sequence for the fuzzy clustering method is encoded by the short length peptides and the combination of functional domain models.

**Results and conclusions:**

With the fuzzy clustering method, all plant CPP transcription factors are grouped into two subfamilies. A systems approach, including Expressed Sequence Tag analysis, evolutionary analysis, protein-protein interaction network analysis and co-expression analysis, is employed to validate the clustering results, the results of which also indicates that the transcription factors from different subfamilies show uncorrelated responses.

## Background

Transcription factors are important in gene expression regulation. Some families of transcription factors have been studied intensively. The CPP (cystein-rich polycomb-like protein) family, in which genes typically have two Cys-rich domains (CXC), however, has not received enough attention. Although there are only a small number of them, the members of this family are widely present in plants and animals.

Two genes in the plant CPP family, TSO1 in Arabidopsis thaliana and CPP1 in soybeans, have been studied previously. TSO1 is highly expressed in flowers, where it is accumulated to the highest level in developing ovules and microspores [1-5]. The Δtso1 mutation results in the loss of the control on directional cellular expansion and the coordination of adjacent cell growth, as well as the defects in karyokinesis and cytokinesis. CPP1 has been demonstrated relating to the regulation of the expression of the soybean leghemoglobin gene Gmlbc3 [6]. The CPP1 gene is induced late in nodule development and its expression is confined to the distal part of the central infected tissue of the nodule. The functions of these two proteins, despite belonging to the same family, are different in * A. thaliana* and Soybeans.

CXC domains are highly conserved in CPP genes. In TSO1 and CPP1, two CXC domains, which have consensus sequence CXCX4CX3YCXCX6CX3CXCX2C, are separated by a region, which contains a variable length sequence with a short conserved sequence RNPXAFXPK[5]. Hauser etc. suggested that the high conservation in the domains and inter-domain regions indicates that the transcription factors in this family could bind to DNA via their CXC domains [1]. This hypothesis has been shown in human; the experiment demonstrated the CXC domain in LIN54 gene in human can bind to a specific DNA sequence CDE-CHR [7].

Though all transcription factors in CPP families have one or two CXC domains, we hypothesize that they have different functions and can be further grouped into subfamilies with similar functions. To test the hypothesis, a fuzzy clustering method with a newly developed feature vector is applied to the protein sequences of all plant CPP transcription factors. A systems approach, including Expressed Sequence Tag (EST) analysis, evolutionary analysis, protein-protein interaction network analysis and co-expression analysis, has been employed to verify the clustering result and to understand the functions of the subfamilies. The results show that the transcription factors in the CPP family can be further grouped into two subfamilies, and they might bind with different DNA sequences and play various regulation roles.

## Results and discussion

### Clustering of CPP family

The total of 111 plant transcription factor proteins in the CPP family are grouped using the fuzzy clustering method. The various numbers of clusters, such as 2, 3, 4, 8 and 50 *etc*. are tested with different membership exponent, including *r *= 1.5, 2, 2.5, 5, 7.5, 8, 8.1, 8.2, 8.5, 9, and 10. When all CPP proteins are clustered into 2 groups with membership exponent *r *= 8.2, the clustering method has the maximal silhouette for each cluster. As a result, the genes are classified into two groups, with 67 and 44 genes, respectively, and referred to as Group I and II in this manuscript. There are 4 genes in *A. thaliana*, including AT3G04850, AT3G22760, AT3G22780, AT4G14770, in Group I, and also 4 genes, AT3G16160, AT2G20110, AT4G29000, and AT5G25790, in Group II. In Group I, there are 10 rice genes and 10 maize genes, and in Group II there are13 rice genes and 9 maize genes. The genes in the same group might share similar functions because of the similar sequence profiles. For instance, AT3G22780 (TSO1) and AT3G22760 (SOL1) are both clustered in Group I, and they share the same function that controls flower tissue development. The function has been validated in experiments; mutations of these two proteins causes plants to not form normal flowers and the cell division loses direction [2-5,8]. Fuzzy clustering, because of the fuzzy logic used, can assign a member to more than one cluster to reflect the fact that one protein may have gradual evolution relationship with the other proteins from different groups. However, all transcription factors in the plant CPP family are classified into two complete groups; there is no common member for these two clusters. This indicates that proteins from different groups are highly distinguishable in protein sequences, and hence, reflecting distinct functions. This discovery is further verified at the sub-sequence and full-length levels by EST analysis and phylogenetic analysis. The protein-protein interaction network analysis and co-expression analysis both validate the independence of transcription factors in the two groups.

### EST analysis

ESTs, short sub-sequence of cDNA sequences, are used for gene discovery and gene sequence determination [9]. The total of 111 DNA sequences of transcription factors in the plant CPP family are scanned against the EST databases. The numbers of aligned ESTs are shown in Table 1. With the dbEST database of NCBI, a total of 810 ESTs are aligned to 57 genes in Group I with E-values <10^-5^, 516 of which are aligned to multiple positions in different or same genes, and 125 ESTs have unique copies. These ESTs are called the redundant and unique ESTs, respectively. The rest of 10 genes in Group I do not have aligned ESTs. The total number of ESTs for the 38 genes in Group II is 384 and the numbers of redundant and unique ESTs are 290 and 94, respectively. Interestingly, genes in the two groups have significantly different sets of ESTs; there are only 165 (31.9%) redundant ESTs, and 5 (4.0%) unique ESTs are common in the two groups. For the CPP genes in Arabidopsis, including *A. thaliana* and *A. lyrata*, Rice, or Maize, EST analysis shows similar results. There are 48 and 26 redundant ESTs, respectively, for Arabidopsis genes in Groups I and II, but only 8 ESTs are shared by the two groups. There are no unique ESTs shared by the two groups in Arabidopsis. For rice genes, 137 and 106 redundant ESTs for Groups I and II have only 24 (17.5%) identical ESTs while only 4 unique ESTs are common in the 77 and 20 ESTs for Groups I and II. The number of common redundant ESTs for maize genes is 107, which is a little high, but it is still only about 40% of the total number of redundant ESTs aligned to the genes in two different groups. However, there is no common EST for the 12 and 50 unique ESTs for Groups I and II. Using the EST data from PlantGDB [10], the results are similar as those obtained with the dbEST database. The two groups have totally 551 and 245 redundant ESTs, and among them, 158 identical ESTs. For unique ESTs, there are only 3 common ESTs between 106 and 148 ESTs. These results support that all plant CPP genes can be categorized into two groups and also indicate that genes from the two groups have significantly different compositions of short motifs.

**Table 1:** EST profiles of two groups

	Group I	Group II	Common
NCBI			
Total genes	57	38	
Redundant	516	294	165
Unique	125	90	5
Arabidopsis	8	6	
Redundant	48	26	8
Unique	20	4	0
Rice genes	10	13	
Redundant	137	106	24
Unique	77	20	4
Maize genes	10	9	
Redundant	265	130	107
Unique	12	50	0
PlantGMD			
Total genes	57	38	
Redundant	551	245	158
Unique	106	148	3
Arabidopsis genes	8	6	
Redundant	35	22	5
Unique	23	2	0
Rice genes	10	13	
Redundant	106	53	9
Unique	41	8	0
Maize genes	10	9	
Redundant	355	148	128
Unique	22	124	2

### Phylogenetic analysis

**Figure 1:**
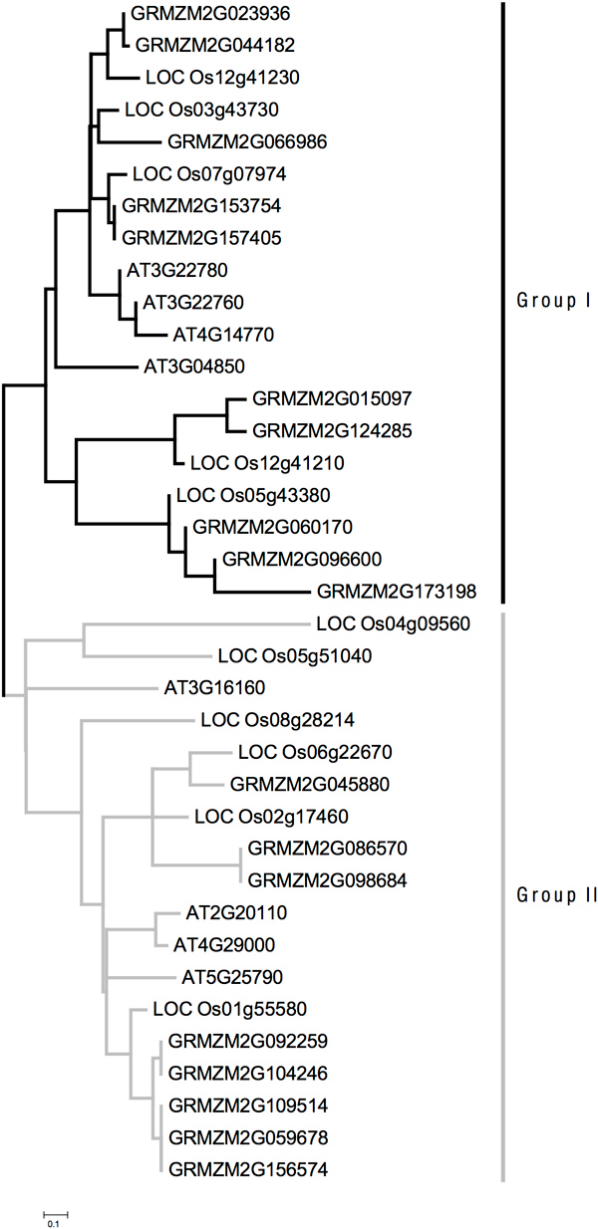
**Phylogenetic tree for genes in *A***. thaliana, rice and maize.

Besides the study of CPP genes at the sub-sequence level with EST analysis, the full-length protein sequences of plant transcription factors in CPP family are analyzed with multiple sequence alignment. The total of 111 proteins are too large to have an efficient and accurate multiple sequence alignment. Therefore, all CPP proteins in *A. thaliana*, *O. sativa Japonica*, and *Z. mays *are collected to study their evolution because these genes have been studied with ESTs analysis, and hence, are good reference for comparison. The Maximum-Likelihood phylogenetic tree is constructed using the alignment of full-length protein sequences with PhyML[11]. The phylogenetic tree of CPP proteins in *A. thaliana*, *O. sativa Japonica *and *Z. mays *is shown in Figure 1. The phylogenetic tree shows that these genes in two different groups distribute into two branches in the tree. The evolution distances among genes in Group I are smaller than those for Group II. The result of phylogenetic analysis agrees with the conclusion of fuzzy clustering.

**Figure 2:**
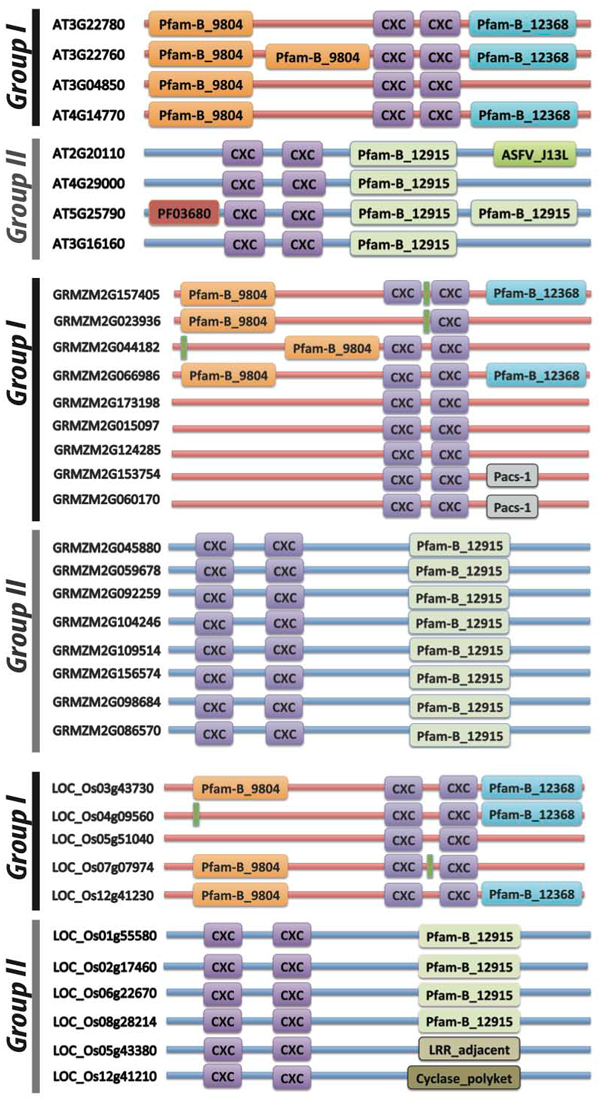
**The diagram of domain distribution of CPP proteins in plants**.

**Figure 3:**
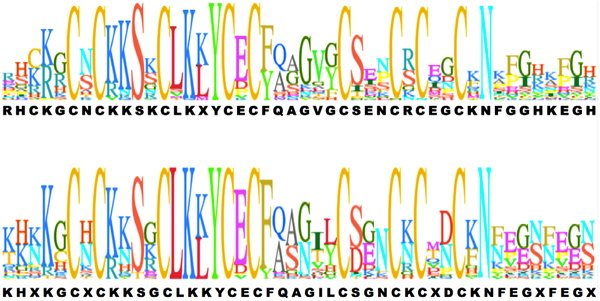
**The logo of DXD domains for group I (upper) and group II (bottom)**.

All plant genes in the CPP family have two tandem CXC domains, but in the Group I, the CXC domains are most often located on in the C-terminal tails of the proteins, while the CXC domains are in the N-terminal parts of genes in the Group II. The diagram of domains in the CPP proteins is shown in Figure 2. The domain models in each protein are obtained from the Pfam database[12]. The average length of proteins in Group I and II are 548 and 411 amino acids, respectively, and the length of a CXC domain is about 46 amino acids. The two conserved CXC domains are a small part of the full-length protein sequences, but the locations of the two CXC domains in the proteins are important for clustering. Other domains are also conserved in Groups I and II, respectively. For example, Pfam-B_9804, a function-unknown domain, appears in the N-terminal parts of many proteins in Group I, while Pfam-B_12915 in the C-terminal parts of proteins in Group II. The CXC domains in two different groups are also compared to each other. The consensus sequence of the CXC domains in Group I is very similar to that in Group II. The logos of consensus sequences are shown in Figure 3. The conserved Cysteins are identical, and most residues in the consensus sequences are the same. However, there are some differences between the CXC domains in genes from two groups. For instance, five residues in the CXC domain of Group II are not conserved, but the corresponding residues in Group I CXC domain are still conserved to certain amino acids. In Group II CXC domains, there are more negative charged residues than those in Group I. The percentages of acidic, basic, and hydrophobic amino acid residues in the CXC domains of proteins in the two groups are shown in Table 2. The average percentage of acidic amino acid residues in the CXC domains for Group I is 5.13% while for Group II it is 8.77%. The percentage of basic and hydrophobic amino acid residues are similar between the two groups.

**Table 2:** The Occurring Frequencies Of Amino Acids In CXC Domains

Group I	Acid (%)	Basic (%)	Hydrophobic (%)
AT3G04850	3.24	23.92	19.57
AT3G22760	4.35	23.92	28.26
AT3G22780	6.53	22.83	23.92
AT4G14770	6.34	22.77	25.07
LOC_Os03g43730	7.61	23.92	19.57
LOC_Os04g09560	3.26	9.79	13.05
LOC_Os05g43380	5.44	20.66	22.83
LOC_Os05g51040	4.35	18.48	26.09
LOC_Os07g07974	5.44	23.92	19.57
LOC_Os12g41230	4.35	20.66	20.66
GRMZM2G015097	6.52	21.74	19.57
GRMZM2G023936	2.18	8.70	9.79
GRMZM2G044182	4.35	21.74	19.57
GRMZM2G066986	2.17	25.00	20.65
GRMZM2G096600	7.11	20.04	23.30
GRMZM2G124285	6.52	21.74	18.48
GRMZM2G157405	5.44	23.92	21.74
GRMZM2G173198	4.35	9.79	7.61
GRMZM2G060170	7.61	19.57	19.57
GRMZM2G153754	5.44	23.92	21.74
Mean	5.14	20.35	20.03
Group II	Acid (%)	Basic (%)	Hydrophobic (%)
AT2G20110	9.88	18.65	20.90
AT3G16160	11.02	22.01	23.02
AT4G29000	9.88	18.65	22.22
AT5G25790	8.77	20.87	18.48
LOC_Os01g55580	7.66	20.87	20.90
LOC_Os02g17460	9.88	25.29	19.81
LOC_Os06g22670	7.53	19.10	24.57
LOC_Os08g28214	5.48	24.13	18.70
LOC_Os12g41210	6.52	22.83	18.48
GRMZM2G045880	9.91	20.85	19.79
GRMZM2G059678	9.88	17.54	17.57
GRMZM2G086570	6.60	23.02	18.72
GRMZM2G092259	9.88	18.65	16.45
GRMZM2G098684	6.60	23.02	18.72
GRMZM2G104246	9.88	18.65	16.45
GRMZM2G109514	9.89	17.54	17.57
GRMZM2G156574	9.88	17.54	17.57
Mean	8.77	20.54	19.41

The CXC domain of the human CPP gene, LIN54, is evolutionarily conserved, and forms a Helix-Coil-Helix secondary structure. The experimental results show that the CXC domain in LIN54 can bind to a specific DNA sequence CDE-CHR (GCGCGG---GTTTGAA), and LIN54 regulates cell cycle [7]. The homological protein in *Drosophila melanogaster *is *Tombola *which binds to the interaction partner *Aly *[8]. LIN54 has many homologous genes in animals, but it shows low similarity with plant CPP genes [7,8]. For the CXC domain only, this DNA binding domain in LIN54 is more homological with those of the CPP proteins in Group II than those in Group I. For example, the CXC domain of gene AT3G22780 in Group I has 56% similarity with that of LIN54 while AT2G20110 in Group II has 60% similarity. Though the difference in the two kinds of CXC domains is not very large, whether the two CXC domains have the same DNA binding sequence or not needs additional experiments to identify.

### Proteins of CCP genes in protein-protein interaction networks

Sequence level analysis shows many differences between the protein sequences from the two different groups, which implies that the transcription factors from the two groups may have different functions. A systems approach is employed to study their properties in various biological networks. Since, as a model organism of plants, only *A. thaliana* has been broadly studied, we focus on 8 CPP genes in *A. thaliana* with the systems-biological analysis. They firstly are mapped to the protein-protein interaction network of *A. thaliana*, and their interaction neighbors are collected. Only one interaction pair is found among proteins in Group I, i.e. AT3G22760 and AT3G22780, which are related to flower development [1-4]. Network analysis show that CPP proteins in Group I do not have one non-CPP neighbor. The localization prediction shows that all proteins in Group I and their neighbors are sorted into nucleus and cytoplasm. Like Group I, there are only interactions among Group II CPP proteins, i.e. the interactions between AT2G20110 and AT4G29000. However, a CPP protein in Group II, AT2G20110, has interactions with other 6 non-CPP proteins: AT5G21274, AT3G43810, AT5G37780, AT3G51920, AT1G66410, and AT2G41090. According to the Gene Oncology annotation [13], AT5G37780 and AT1G66410 are the calcium and protein binding signal proteins. Although there are not many data for the protein interactions, one may get a clue that there is only a small chance to have interactions between two CPP proteins from two different groups. The existing interactions among the CPP proteins are only for proteins from the same group.

### CCP co-expression genes and tissue specificity

Using PlaNet (Plant co-expression network browser), the co-expressed genes among the CPP genes in *A. thaliana *are identified. Like found with the protein-protein interaction network, there are not many co-expressed genes discovered among the CPP genes, but genes in the same group more tend to have similar expression profiles. The lists of co-expressed genes for the two different groups do not have any common genes. In Group I, AT3G22780 has many co-expressed genes; the expression profiles of 16 genes have large correlations with AT3G22780's expression profile. These 16 genes are related to cell division. In Group II, AT3G16160 has the most co-expression partners, and they are related to DNA synthesis. CPP genes in *A. thaliana *are also queried to the gene expression database, PRINTs[14], for their expression profiles in various tissues. The results show that 4 genes in Group I have high level expression at flower stage, while the other 4 genes in Group II have low expression levels in any tissue. The tissue specificity of genes in Group I agrees with the previous work, which showed genes, AT3G22780 and AT3G22760, control flower tissues development [2-5,8]. The gene expression profile analysis shows that genes in two CPP groups often have different characteristics, which may indicate that they belong to different subfamilies.

## Conclusions

Transcription factors having CXC domain are grouped together as the CPP family, but they do not necessarily have the same function. Using the fuzzy clustering method, plant CPP proteins can be further clustered into two groups. The feature vectors for the fuzzy clustering method are constructed by quantifying the occurrence frequencies of the short length peptides and the combination of Pfam domain models. Based on the results of EST analysis, evolutionary analysis, protein-protein interaction network analysis, and co-expression analysis, transcription factors from the two groups show distinct characteristics. The systems approach supports the conclusion that plant CPP transcription factors belong to two different subfamilies. This work showcases an example of the biological application of fuzzy clustering.

## Methods

### Fuzzy clustering

To conduct the clustering, each protein sequence is quantified as a feature vector. For each protein sequence, a feature vector is encoded with the normalized occurrence frequency of short peptides and the combination of functional domain model. The lengths of short peptides used are 1, 2, 3, and 4 amino acids. The normalized occurrence frequencies of *n*-peptide are calculated as

(1) $$A_{i}=w\frac{f_{i}}{\sum_{j=1}^{n}f_{j}}$$

where *w=*10*^n^*, (*n *= 1, 2, 3, or 4), is the weight factor, and *f_i _*is the frequency of a type of peptide occurring in a given protein. For an *n*-peptide, there are 20*^n ^*bits in the feature vector. The combination of functional domains is determined by Pfam [12] domain models. For a given protein, its protein sequence is scanned against the Pfam domain database [12] with the cutoff E-value = 1, and the numbers of different type domains are saved in the feature vector. A weight of 100 is applied on the raw value for the domain attribute in the feature vector.

Fuzzy clustering is a soft clustering method, which considers a point as a degree of belonging to clusters, as in fuzzy logic. This means fuzzy clustering method can assign a member to more than one groups and associate the member with a set of membership levels. The fuzzy clustering method is suitable to classify biological items that have gradual evolution relationship and cannot be divided into two completely different groups. The fuzzy clustering algorithms have been thoroughly studied and overviewed before [15]. In this manuscript, the fuzzy clustering function, *fanny()*, in the package of cluster (Version 1.14.2) of R is used. The algorithm is similar to fuzzy C-means clustering, and was described by Kaufman and Rousseeuw[16]. For given *n *points *X*=*x_1_*, *x*_2_, ..., *x_n_*, the fuzzy C-means clustering algorithm groups them into a collection of *k *fuzzy clusters, *C*=*c_1_, c_2_, ..., c_k _*with a membership *u_iv _*assigned for a point *x_i _*to a cluster *c_v_*. Like the *k*-means clustering algorithm, fuzzy C-means clustering aims to minimize the average distance of members to the centroid of each cluster. The objective function is

(2) $$min:\overset{k}{\underset{v=1}{\sum }}\overset{}{\underset{ij}{\sum }}\frac{u_{iv}^{r}u_{jv}^{r}d\left(i,j\right)}{2\overset{}{\underset{j}{\sum }}u_{jv}^{r}}$$

where *d(i,j) *is the dissimilarity between points *i *and *j*, and *r *is the membership exponent, which determines the level of cluster fuzziness. The value of *r *is larger than 1, and the default value is 2. The iteration to minimize the objective function is similar to the *k*-means clustering algorithm. This fuzzy clustering function, *fanny()*, is more robust and provides the silhouette plot for assessment.

Silhouette is a measure of clustering, and is used to determine the quality of clusters [17]. Silhouette is defined as,

(3) $$S_{i}=\frac{b_{i}-a_{i}}{max\left\{a_{i},b_{i}\right\}}$$

where *S_i _*is the *i-*th cluster silhouette, *a_i _*is the average dissimilarity of the *i-*th cluster with all other clusters, *b_i _*is lowest average dissimilarity to any other cluster, except the *i-*th cluster. As the definition, the silhouette is between -1 and 1. If silhouettes are close to 1, data are appropriately clustered. The silhouette is used as the major assessment, and the number of clusters and the membership exponent, *r*, are changed to maximize the value of silhouette.

### CPP protein sequences

A total of 133 CPP genes in 16 plants are obtained from the database of PlnTFDB (http://plntfdb.bio.uni-potsdam.de) [18]. All the 133 protein sequences are screened against the RefSeq [19] in NCBI with BLAST [20], and 111 DNA sequences are obtained. In this manuscript, these 111 genes are used to study the plant CPP family. The protein sequences of CPP-like genes in other non-plant species are obtained from the Pfam database [12]. The number of the CPP family from other eukaryote species is 214, which are from 71 species.

### Expression profiles *in silico*

The expression profiles of CPP genes are estimated by the EST numbers that are obtained by searching against the dbEST database (http://www.ncbi.nlm.nih.gov/dbEST). MEGABLAST is used to search in dbEST database with the cutoff of E-value = 10^-10^. The EST data from PlantGDB (http://www.plantgdb.org) [10] is also used to study the CPP genes.

### Phylogenetic Analysis

Multiple sequence alignment is conducted using ClustalW [21]. Maximum-Likelihood phylo-genetic tree is constructed by PhyML program [11] with the following parameters: start tree, BioNJ [22]; tree topology research, Nearest Neighbor Interchanges (NNIs) [23]; model of amino acids substitution, BLOSUM62 [24]. The tree reliability is estimated by aLRT (approximate Likelihood Ratio Test) [25] of PhyML, with SH-like statistic method [11].

### Protein-protein interaction network and expression profiles

*Arabidopsis *protein-protein interaction networks are constructed with four different resources. They are AtPIN (http://bioinfo.esalq.usp.br/atpin/atpin.pl) [26], TAIR interactome (http://www.mmnt.net/db/0/0/ftp.arabidopsis.org/Proteins/Protein_interaction_data/Interactome2.0), AtPID (http://www.megabionet.org/atpid/webfile/) [27], and athPPI (http://bioinformatics.psb.ugent.be/supplementary_data/stbod/athPPI/site.php) [28,29]. The gene expression profiles are obtained from PlaNet (http://aranet.mpimp-golm.mpg.de/) [30], and the tissue specificity data are gathered from the PRINTs database (http://www.bioinf.manchester.ac.uk/dbbrowser/PRINTS/index.php) [14].

## Competing Interests

The authors declare that they have no competing interests.

## Authors' contribution

TL designed the study and implemented the algorithm. TL and YD prepared the data. CZ supervised the whole project and drafted the manuscript.

## Declarations

The work is supported by funding under CZ's startup funds from University of Nebraska, Lincoln, NE.

This article has been published as part of BMC Bioinformatics Volume 14 Supplement 13, 2013: Selected articles from the 9th Annual Biotechnology and Bioinformatics Symposium (BIOT 2012). The full contents of the supplement are available online at http://www.biomedcentral.com/bmcbioinformatics/supplements/14/S13
